# Physicians’ Visiting List List

**Published:** 1875-11

**Authors:** 


The Physicians' Visiting List for 1876. Philadelphia: Lindsay & Blakiston. Leather.

This neat and well arranged Visiting List for physicians, comes to hand, with its same popularity as before. Its already universal use is the best evidence of value.

